# Supplementary material for: Effects of warm-season feeding on yak growth, antioxidant capacity, immune function, and fecal microbiota
Source: Microbiol Spectr. 2025 Jun 23;13(8):e01001-25. doi: 10.1128/spectrum.01001-25 (PMC12323587; doi:10.1128/spectrum.01001-25)
Supplement: Supplemental figures — Figures S1 to S3. [file spectrum.01001-25-s0001.docx]

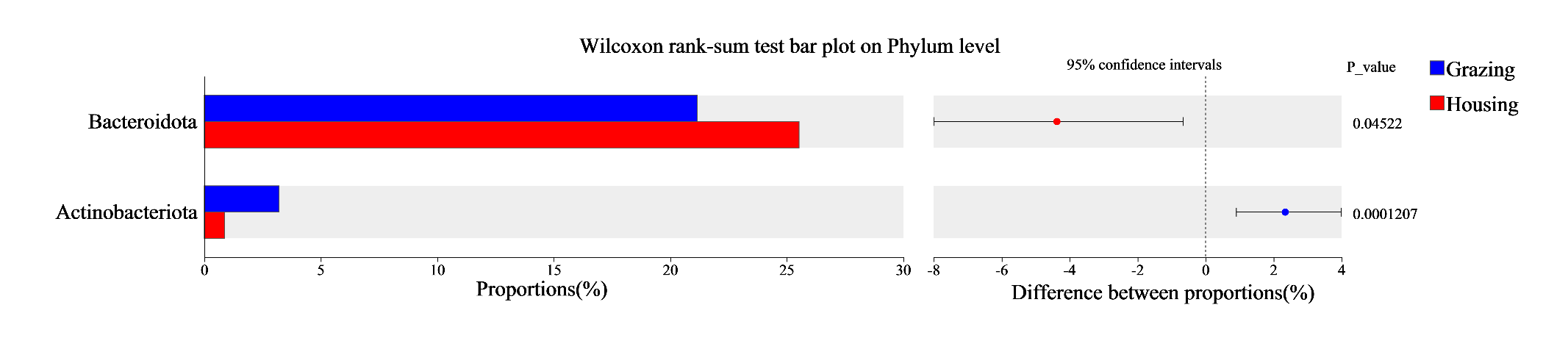


**Supplementary Materials Figure 1** Bar plot of differential microorganisms at the phylum level.


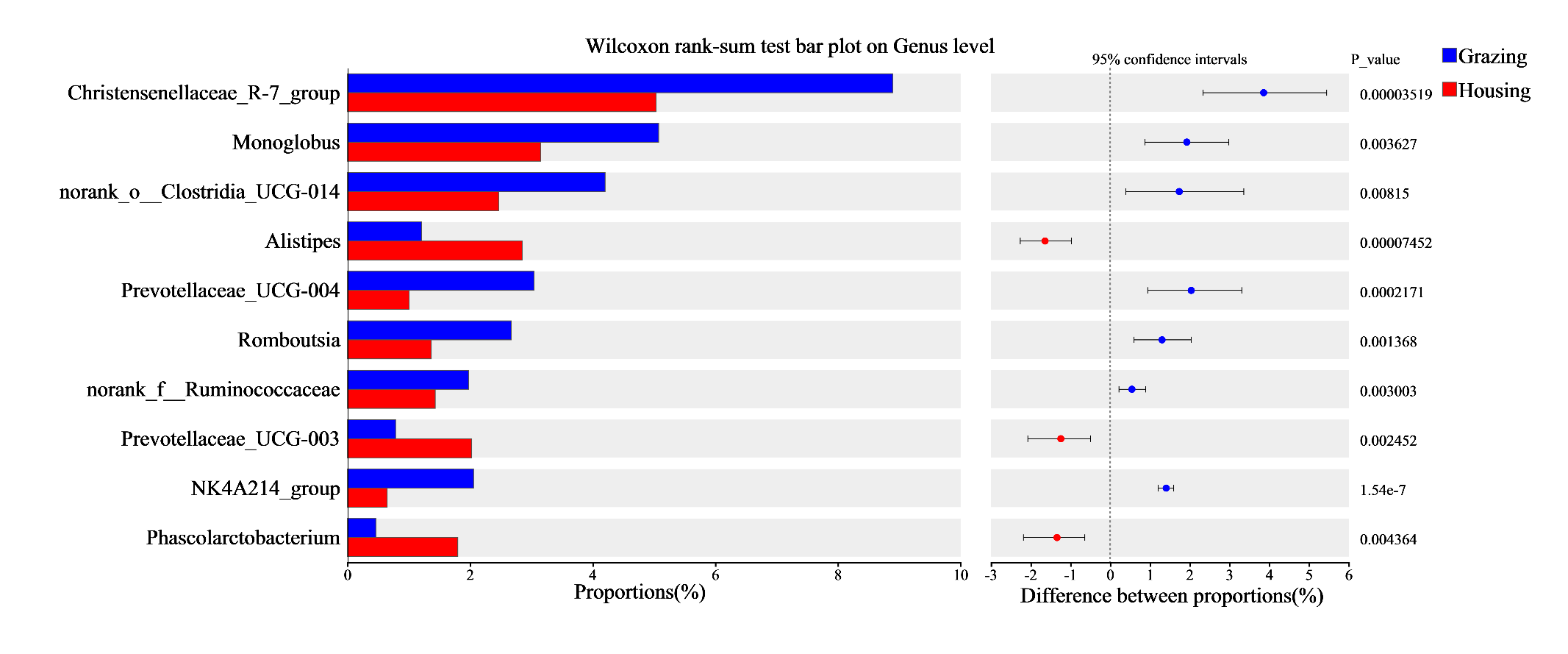


**Supplementary Materials Figure 2** Bar plot of differential microorganisms at genus level.


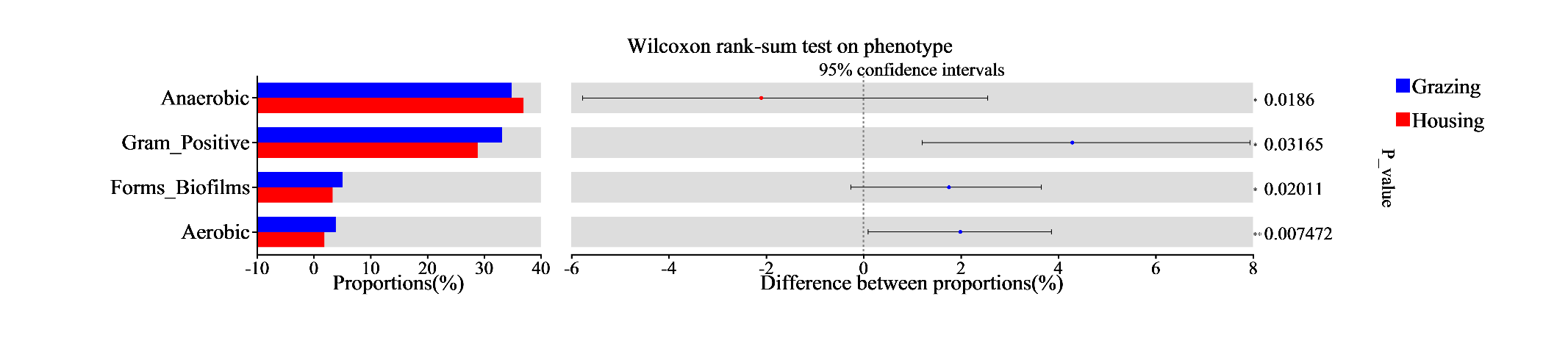


**Supplementary Materials Figure 3** Bar Chart of BugBase Difference Test.
